# Evaluation of D2-plus radical resection for gastric cancer with pyloric invasion

**DOI:** 10.1186/s12893-019-0605-6

**Published:** 2019-11-20

**Authors:** Zhi-yuan Xu, Can Hu, Shangqi Chen, Yi-an Du, Ling Huang, Peng-fei Yu, Li-jing Wang, Xiang-dong Cheng

**Affiliations:** 1 Department of Abdominal Surgery, Zhejiang Cancer Hospital, Hangzhou, Zhejiang Province China; 2 Zhejiang Chinese Medical University, Hangzhou, Zhejiang Province China; 3 Department of Ultrasonics, The Zhejiang Cancer Hospital, Hangzhou, Zhejiang Province China

**Keywords:** Advanced gastric cancer, Pylorus invasion, D2-plus lymphadenectomy

## Abstract

**Background:**

The optimal lymphadenectomy for gastric cancer (GC) with pyloric invasion is controversial because the pattern of lymph node metastasis is different from that of distal GC. The rate of lymph node metastasis into the posterior area of the pancreatic head and hepatoduodenal ligament is high. This study evaluated the estimated benefit of radical gastrectomy with D2-plus lymphadenectomy in patients with pyloric invasion.

**Methods:**

All patients with GC invading the pylorus who underwent curative surgical resection with D2-plus lymphadenectomy between February 2013 and September 2015 were enrolled in the study. The index of estimated benefit from lymph node dissection (IEBLD) was calculated by multiplying the incidence of metastasis to each lymph node station by the 3-year overall survival (OS) rate of patients with metastasis to that station.

**Results:**

In total, 128 patients were eligible. The rate of lymph node metastasis and the 3-year OS rate (and IEBLD) of the patients with metastasis to lymph nodes were 14.3 and 44.4% (5.56) for No. 8p, 10.9 and 35.7% (3.89) for No. 12b, 9.5 and 33.3% (3.13) for No. 12p, 18.8 and 54.2% (10.19) for No. 13, and 21.8 and 53.6% (11.68) for No. 14v, respectively.

**Conclusions:**

In radical gastrectomy for GC with pyloric invasion, some survival benefit was observed with dissection of the No. 13 and No. 14 lymph nodes, but there was no survival benefit with dissection of the No. 8p lymph nodes. The No. 12b and No. 12p lymph nodes may be better to dissect in cT3 GC patients with pyloric invasion.

**Trial registration:**

http://ClinicalTrials.gov Identifier: NCT01836991. Date of registration: April 17, 2013.

## Introduction

Gastric cancer (GC) is a common malignancy and has been estimated to account for one-third of cancer-related deaths [1]. Because GC is asymptomatic at the early stage of the disease, it is frequently diagnosed in later stages in China. Surgery is the optimal treatment for patients with GC and provides the best chance of long-term survival. As the lymphatic route is the major pathway for GC metastasis, radical gastrectomy with sufficient lymph node dissection is the key factor for the surgical treatment of GC. Since the 15-year follow-up results of the Dutch trial [2] showed that patients treated with D2 lymphadenectomy had a lower rate of locoregional recurrence than patients treated with D1 lymphadenectomy [3], D2 lymphadenectomy has become the standard treatment for resectable GC worldwide.

However, because of the special location of the pyloric canal, the metastasis pattern and the lymphatic drainage are different in GC with pyloric invasion. A study by Chen et al. [4] showed that the rates of metastasis to the hepatoduodenal ligament and the posterior area of the pancreatic head, including lymph nodes behind the hepatic artery (No. 8p), along the bile duct in the hepatoduodenal ligament (No. 12b), behind the portal vein (PV) in the hepatoduodenal ligament (No. 12p), in the retropancreatic area (No. 13) and along the superior mesenteric vein (SMV) (No. 14v), were high in GC with pyloric invasion. According to the new Japanese Gastric Cancer Association (JGCA), the No. 14v lymph nodes are regarded as regional lymph nodes, and the No. 13 lymph nodes are regarded as regional lymph nodes in GC with duodenal invasion. However, the No. 8p, 12b, 12p, and 13 lymph nodes are not included in D2 lymphadenectomy for lower stomach cancer.

The aim of this study was to evaluate the significance of D2-plus lymphadenectomy including dissection of the second-station lymph nodes in lower stomach cancer (including the No. 14 lymph node) and the No. 8p, 12b, 12p, and 13 lymph nodes for GC with pyloric invasion via assessing the incidence of metastasis and patient survival.

## Methods

### Patients

This study was conducted as a prospective multi-institutional trial involving 2 institutions. The protocol of this study was approved by the Protocol Review Committee of the Zhejiang Cancer Hospital and First Affiliated Hospital of Zhejiang Chinese Medical University. All enrolled patients provided written informed consent and showed that their permission to join in the study before study entry. This trial was registered with the ClinicalTrials network (http://www.clinicaltrial.gov) as NCT01836991. The inclusion criteria were as follows: (1) Males and females aged 18 to 70 years old. (2) Preoperative evaluation showing distal gastric cancer with pyloric invasion, ≥T2 or N+ or stage II, IIIA, or IIIB. (3) Karnofsky score ≥ 70, and life expectancy > 6 months. (4) Endoscopic biopsy diagnosis of adenocarcinoma, excluding non-Hodgkin’s lymphoma, leiomyosarcoma and other mesenchymal tumors. (4) Blood and biochemical indicators meeting the following criteria: hemoglobin (Hb) ≥ 9 g/dl; white blood cell (WBC) count≥4000/mm^3^, ≤12,000/mm^3^; platelet (PLT) count≥100,000/mm^3^. (5) Aspartate aminotransferase (GOT) and alanine aminotransferase (GPT) within twice the institutional limit, serum total bilirubin < 1.5 times the upper limit of normal, and serum creatinine < 1.25 times the upper limit of normal. (6) No prior chemotherapy, radiotherapy or biological therapy.

### Surgery

The surgical technique for lymphadenectomy has been previously described in detail [5]. The midline of the upper abdomen was incised, an abdominal exploration was performed, and the tumor location, classification and lymph node metastasis were verified. A sharp dissection was performed at the lateral border of the duodenum such that the duodenum was freed.

**Fig. 1:**
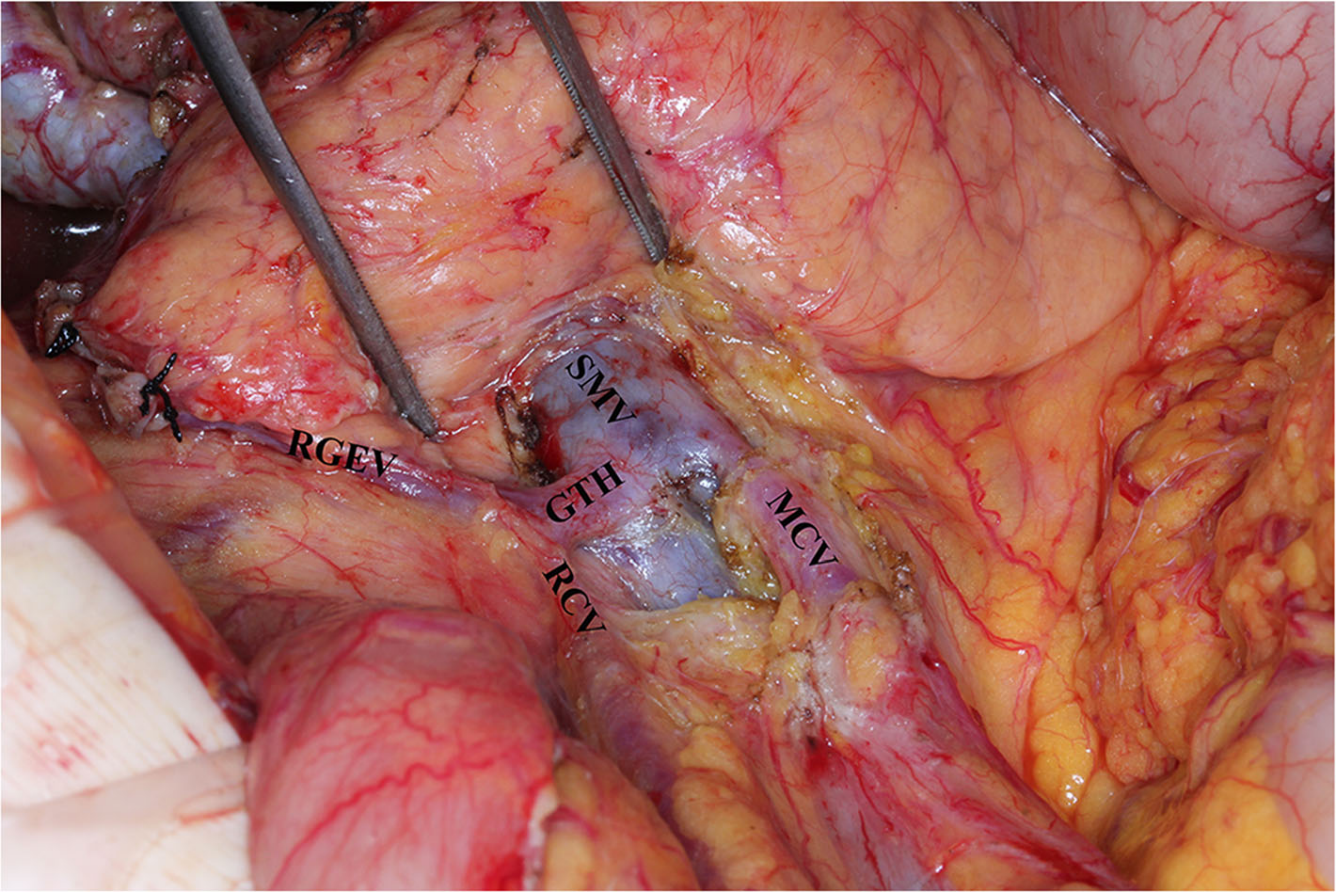
The anatomical structure after the No.14v lymph node dissection. SMV: superior mesenteric vein; MCV: middle colic vein; GTH: gastrointestinal vein trunk; RGEV: right gastroepiploic vein; RCV: right superior colic vein

**Fig. 2:**
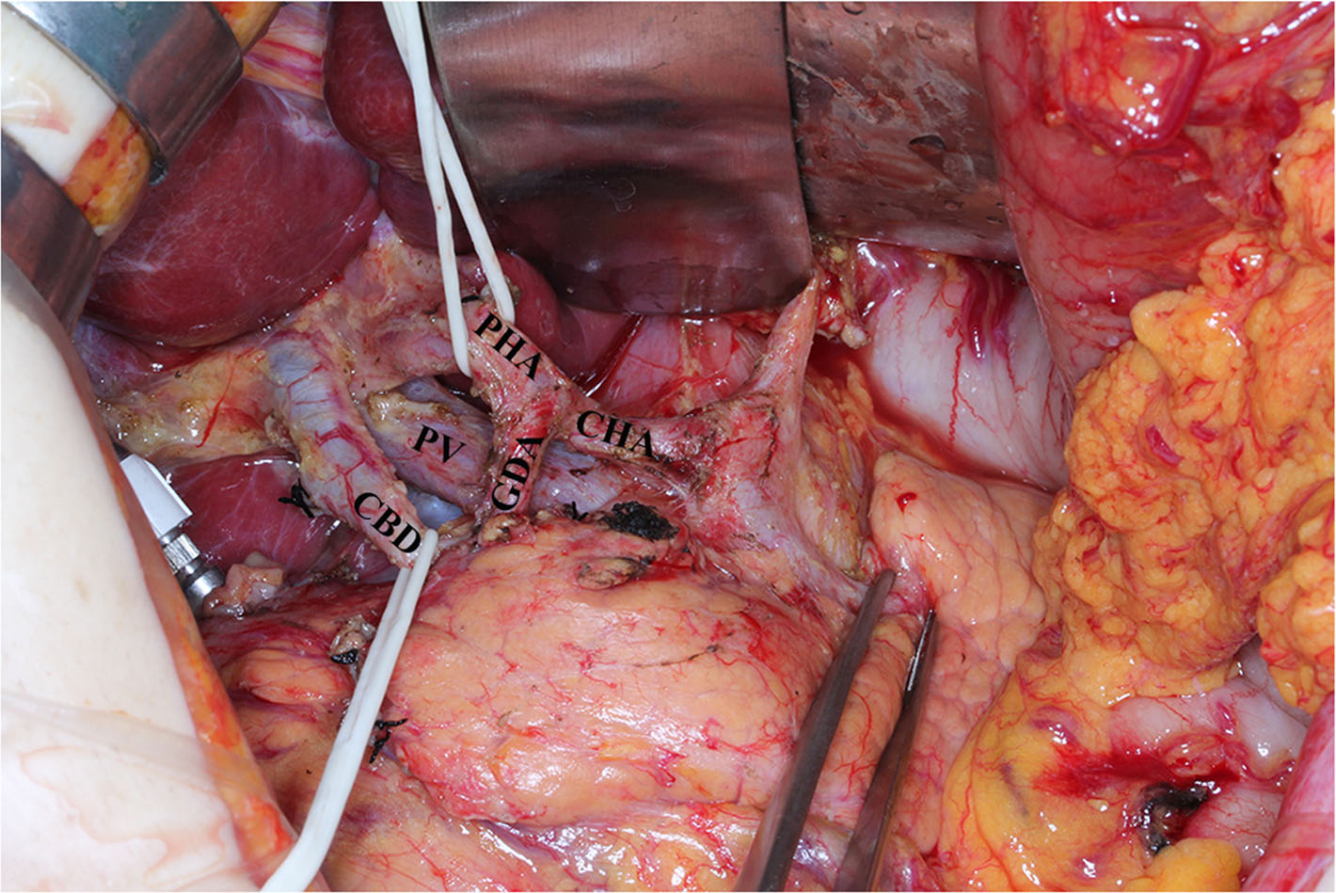
The anatomical structure of hepatoduodenal ligament after lymph lymph nodes dissection. PHA: proper hepatic artery; GDA: gastroduodenal artery; CHA: common hepatic artery; CBD: common bile duct; PV: portal vein

First, the lymph node dissection was performed in the area inferior to the pylorus along the course of the middle colic vein toward the SMV, as well as the gastrointestinal vein trunk and accessory right colic vein. The lymph nodes along the SMV (No. 14v) were dissected (as shown in Fig. 1). The separation was continued toward the pylorus, and the right gastroepiploic blood vessel was cut. The subpyloric lymph nodes (No. 6) were dissected. Then, a separation was made along the superior border of the pancreas, and the left gastroepiploic blood vessel was separated and cut. The splenic artery was revealed, and the lymph nodes along the splenic artery were dissected. The separation was continued toward the left diaphragmatic muscle. The hepatoduodenal ligament was opened, the proper hepatic artery (PHA) and the right gastric artery were separated, and the latter blood vessel was cut. The suprapyloric lymph nodes (No. 5) were dissected. The duodenum was cut with a linear stapler 3 cm below the pylorus, and the stumps were closed with reinforced stitching. The lymph nodes surrounding the PHA (No. 12a) were dissected. Third, the common bile duct (CBD) was separated, and the lymph nodes around the CBD (No. 12b) were dissected. The PV was separated, and the lymph nodes along the PV (No. 12p) were dissected. Then, the separation was continued along the common hepatic artery, and the surrounding lymph nodes (No. 8a and No. 8p) were dissected (as shown in Fig. 2). The left gastric blood vessel was separated and cut. The separation was continued toward the cardia, and the lymph nodes on the right side of the cardia (No. 1) were dissected. Finally, the gastric artery was cut with a linear stapler 5 cm from the tumor, and the distal gastric artery was separated together with the lymph nodes. Finally, Billroth II gastrojejunostomy and Braun anastomosis was performed.

After the surgery, every lymph node was separated from the fresh specimens according to the JGCA 3rd edition criteria [6], and all resected specimens were examined by the same pathology team to assess the extent of residual disease and the disease stage. Surgical complications were assessed according to the Clavien–Dindo classification [7].

### Postoperative evaluation and follow up

The postoperative evaluation has been previously described in detail [8]. And the patients were examined regularly every 3 months during the first year and every 6 months thereafter.

### Evaluation of the therapeutic value of D2-plus lymph node dissection

The IEBLD was proposed by Sasako et al. [9] to evaluate the efficacy of the dissection of each lymph node station. The IEBLD is determined by multiplying the frequency of lymph node metastasis to each station by the 5-year OS rate of patients with metastasis to that station. However, we had only 3-year OS data in this study; thus, the IEBLD was calculated by multiplying the frequency of lymph node metastasis to each station by the 3-year OS rate of patients with metastasis to that station.

### Statistical analysis

Statistical analysis was performed with SPSS 20.0 statistical software. Survival curves were estimated using the Kaplan–Meier method. And the Survival curves in cT3 and cT4a patients were estimated by log-rank test. A *p* value of < 0.05 was considered statistically significant.

## Results

### Patients and lymph nodes

In our group, 128 patients (82 males, 46 females) underwent gastrectomy with D2-plus lymphadenectomy. Among them, the mean body mass index (BMI) of the patients was 23.5 ± 2.1 kg/m^2^, and the median patient age was 61 (range: 37–71) years. All patients underwent subtotal gastrectomy, and 26 patients underwent resection including the duodenum and pancreas. The tumor diameter in 72 patients was greater than 5 cm, while the tumor diameter in 56 patients was less than 5 cm. Lymph node metastasis occurred in 72 patients, and 44 patients showed invasion of the serosa or adjacent organs. The characteristics of the study patients are shown in Table 1.

**Table 1:** Patient characteristics

Characteristic	Value
Number of case	128
BMI (Kg/m2)	23.5 ± 2.1
Sex	
Male	82
Female	46
Age	
< 65	98
≥ 65	30
Tumor diameter	
< 5 cm	56
≥ 5 cm	72
Pancreaticoduodenectomy	26
Pyloric obstruction	
Yes	46
No	82
Duodenal invasion	
Yes	31
No	97
Depth of invasion	
pT2	12
pT3	72
pT4a	37
pT4b	7

### Postoperative complications

Seventeen patients had postoperative complications, including wound infection (2 patients), lymphorrhagia (4 patients), anastomotic stricture (4 patients), pancreatic leakage (3 patients) and anastomotic leakage (4 patients). In all, 11 grade I complications (not requiring special treatment), 2 grade II complications (requiring special treatment, such as a blood transfusion), 4 grade IIIa complications (requiring surgical, endoscopic or radiological intervention without anesthesia), and no grade IIIb or higher complications were reported. All complications were resolved with conservative treatment. The postoperative complications are shown in Table 2.

**Table 2:** Postoperative complications

	*N* = 128
Postoperative complications	17
Incision infection	2
Lymphorrhagia	4
Anastomotic stricture	4
Pancreatic leakage	3
Anastomotic leakage	4

### Therapeutic value of lymph node dissection

The evaluation of each lymph node station is shown in Table 3. Dissection of the No. 8p, 12b, 13, and 14v lymph nodes was completed in all patients, while 126 patients underwent No. 12p lymph node dissection. The rate of lymph node metastasis and the 3-year OS rate (and IEBLD) of the patients with metastasis to lymph nodes were 14.3 and 38.9% (5.56) for No. 8p, 10.9 and 35.7% (3.89) for No. 12b, 9.5 and 33.3% (3.13) for No. 12p, 18.8 and 54.2% (10.19) for No. 13, and 21.8 and 53.6% (11.68) for No. 14v, respectively.

**Table 3:** Frequency of lymph node metastasis and 3-year survival in patients with pyloric invasion

LN station number	Incidence of LN metastasis (%)	3-years OS (%)	Therapeutic value index
1	11.1 (14/126)	57.1	6.3
3	32.0 (41/128)	61.0	19.52
4sa	0 (0/3)	–	–
4sb	4.9 (4/82)	0	0
4d	29.5 (36/122)	55.6	16.4
5	26.5 (34/128)	52.3	13.9
6	51.6 (66/128)	57.6	29.7
7	22.9 (28/128)	57.1	13.1
8a	29.7 (38/128)	47.4	14.1
8p	14.3 (18/128)	38.9	5.56
9	20.3 (26/128)	46.2	9.4
11p	12.5 (16/128)	50	6.25
12a	17.2 (10/128)	60	10.3
12b	10.9 (14/128)	35.7	3.89
12p	9.5 (12/126)	33.3	3.13
13	18.8 (24/128)	54.2	10.19
14v	21.8 (28/128)	53.6	11.68

### Survival of GC patients with or without No. 8p, 12b, 12p, 13 or 14v lymph node metastasis

**Fig. 3:**
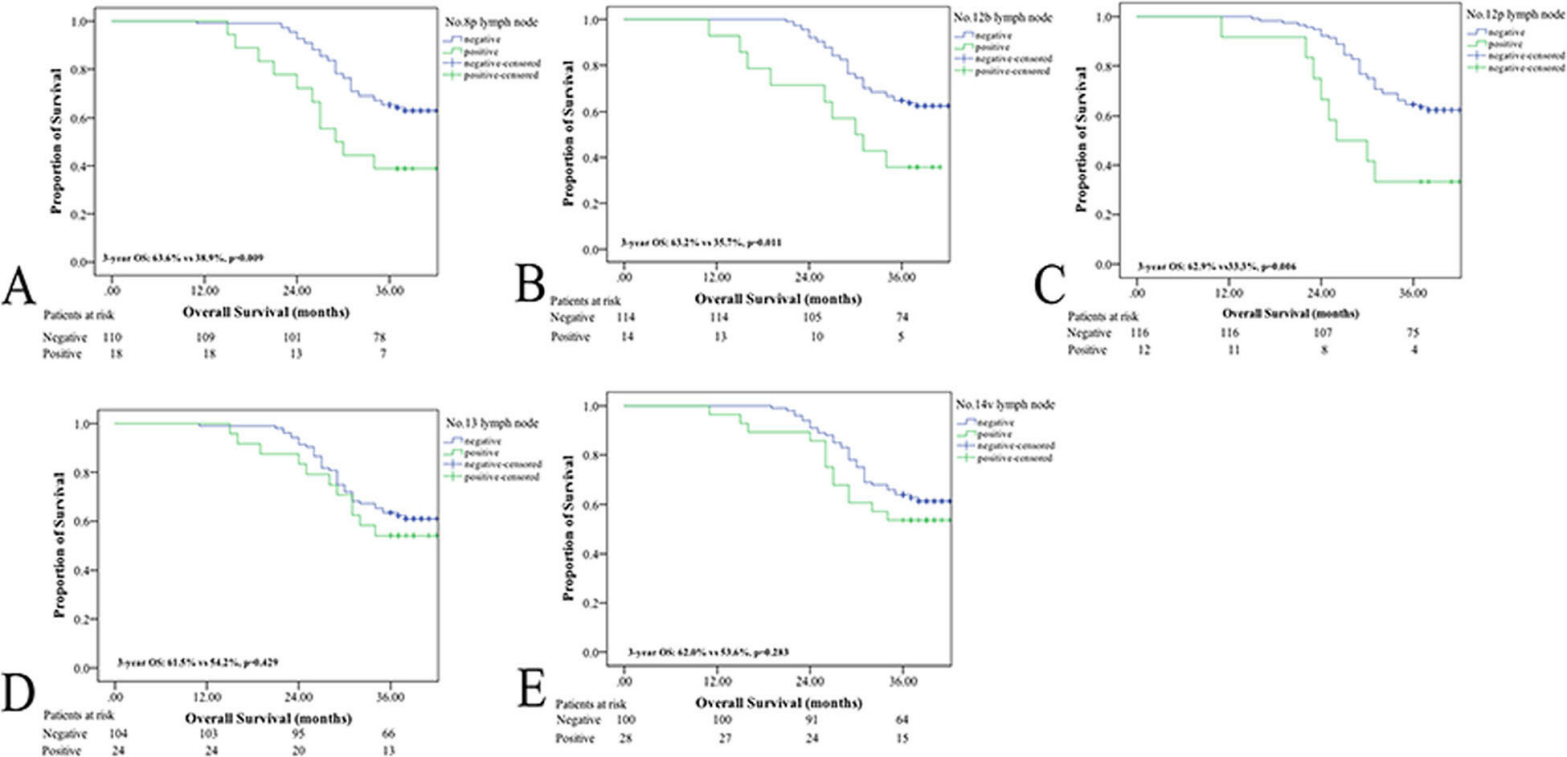
Survival curves for GC patients with or without No.8p, No.12b, No,12p, No.13 and No.14v lymph node metastasis. **a** patients with No.8p metastasis; **b** patients with No.12b metastasis; **c** patients with No.12p metastasis; **d** patients with No.13 metastasis; **e** patients with No.14v metastasis; **f** all patients categorized by cT stage

**Fig. 4:**
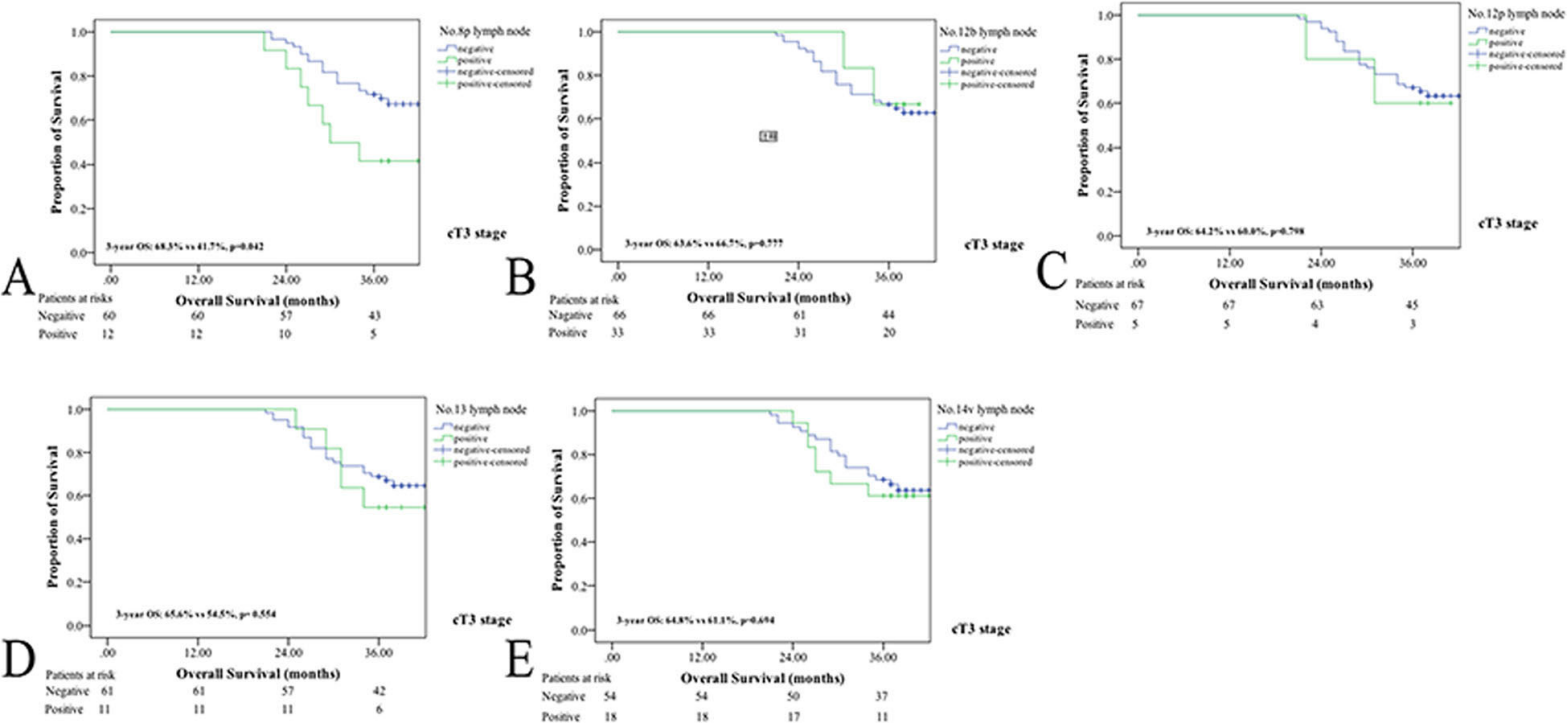
Survival curves for in cT3 stage GC patients with or without No.8p, No.12b, No,12p, No.13 and No.14v lymph node metastasis. **a** patients with No.8p metastasis; **b** patients with No.12b metastasis; **c** patients with No.12p metastasis; **d** patients with No.13 metastasis; **e** patients with No.14v metastasis

**Fig. 5:**
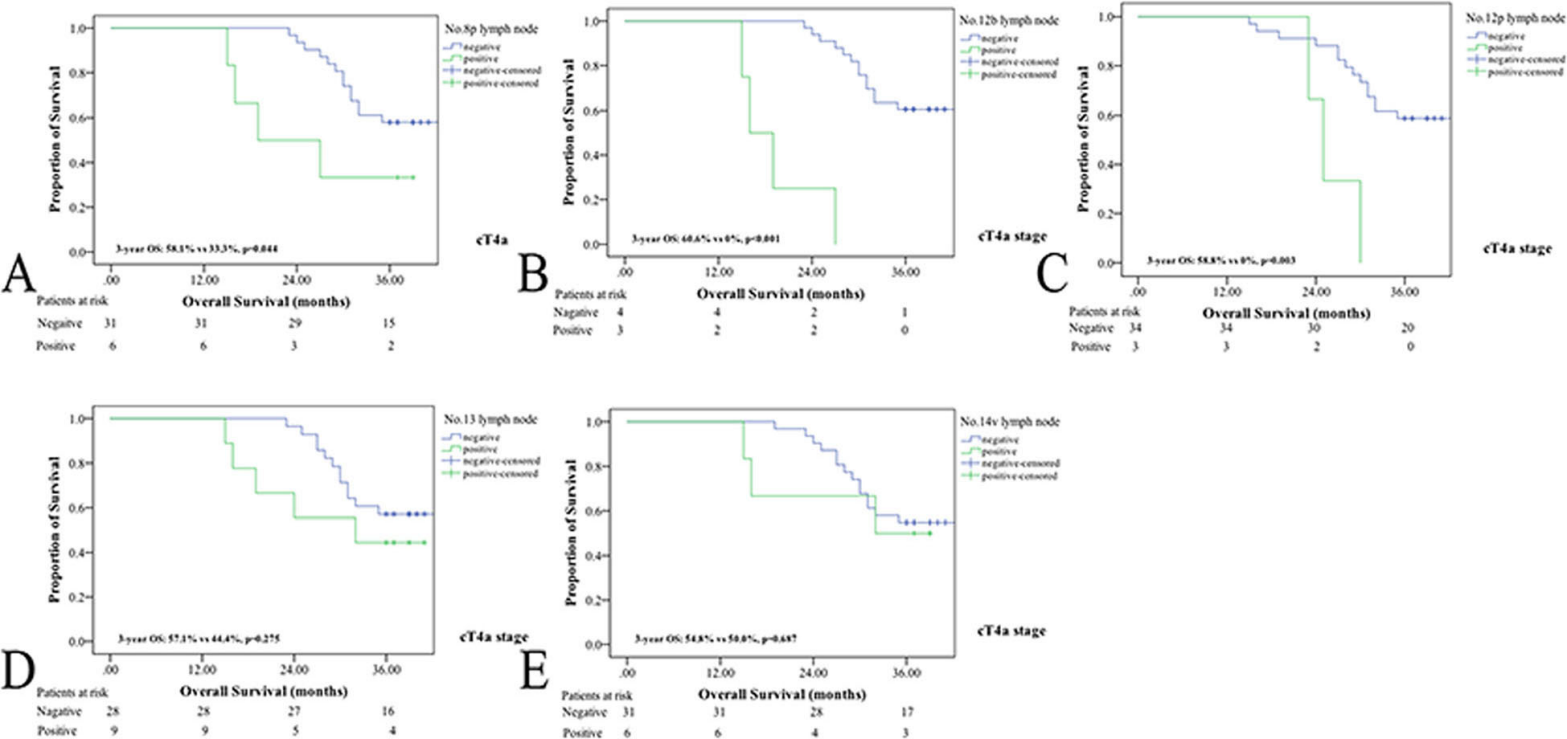
Survival curves for cT4a GC patients with or without No.8p, No.12b, No,12p, No.13 and No.14v lymph node metastasis. **a** patients with No.8p metastasis; **b** patients with No.12b metastasis; **c** patients with No.12p metastasis; **d** patients with No.13 metastasis; **e** patients with No.14v metastasis

In the present study, the 3-year OS rate was significantly higher in GC patients without than with No. 8p, No. 12b or No. 12p lymph node metastasis (3-year OS rate: 63.6% vs 38.9%, *p* = 0.009; 63.2% vs 35.7%, *p* = 0.011; 62.9% vs 33.3%, *p* = 0.006) (as shown in Fig. 3). However, there were no differences in the OS of GC patients with or without No. 13 or No. 14 lymph node metastasis (3-year OS rate: 61.5% vs 54.2%, *p* = 0.429; 62.0% vs 53.6%, *p* = 0.283). Interestingly, the 3-year OS rate was not significantly different between cT3 GC patients with or without No. 12b or No. 12p lymph node metastasis (3-year OS rate: 63.6% vs 66.7%, *p* = 0.777; 64.2% vs 60.0%, *p* = 0.798). Nevertheless, metastasis to the No. 8p, No. 12b, or No. 12p lymph nodes was a negative prognostic factor in cT4 patients (as shown in Figs. 4 and 5).

## Discussion

Goto et al. [10] showed that metastasis to the lymph nodes along the celiac artery was a significant prognostic factor in GC. This study evaluated the effect of additional No. 8p, 12b, 12p, 13, and 14v dissection during D2-plus lymphadenectomy on survival in GC with pyloric invasion. Some of the lymph node stations mentioned above were regarded as distant lymph nodes for lower GC, but the rate of metastasis to these lymph nodes was high in some studies [11].

The No. 8 lymph nodes were divided into anterosuperior lymph nodes (No. 8a) and posterior lymph nodes (No. 8p) along the common hepatic artery. According to the guidelines of the JGCA, the No. 8p nodes are regarded as distant lymph nodes, but some studies have shown that the rate of No. 8p lymph node metastasis is high. It is also controversial whether No. 8p should be dissected in lower 1/3 GC. In addition, a higher rate of No. 8p lymph node metastasis has been reported in lower 1/3 GC than in upper/middle 1/3 GC, and tumor size, tumor location and depth of invasion are reportedly significant factors for No. 8p lymph node metastasis [12]. Chen et al. [4] showed that No. 8a, 3, 6, 7, or 11p lymph node metastasis is a risk factor for No. 8p lymph node metastasis. Furthermore, positive No. 8p lymph nodes is a significant prognostic factor because of the special location near the abdominal aorta. In the present study, we confirmed that the rate of No. 8p (14.3%) lymph node metastasis was higher than that of N2 lymph nodes (Nos. 4sa, 4sb, and 11p), but patients with positive No. 8p lymph nodes had a low 3-year OS rate, which led to the relatively low IEBLD for the No. 8p lymph nodes (5.56). This finding may be similar to the results of the study conducted by Kumagai et al. [11]. Univariate analysis showed that of No. 8p lymph node metastasis increased the risk of poorer OS; however, it was not an independent prognostic factor on multivariate analysis [13]. We could not provide a survival benefit or additional value by dissecting the No. 8p lymph nodes. Furthermore, because of the deep location, dissection of the No. 8p lymph nodes may increase the risk of bleeding, lymphorrhagia and pancreatic trauma. Therefore, it may be not necessary to dissect the No. 8p lymph nodes.

The hepatoduodenal lymph nodes (No. 12) are divided into lymph nodes along the hepatic artery (No. 12a), along the bile duct (No. 12b), and behind the portal vein (No. 12p). The No. 12b and No. 12p lymph nodes are regarded as N3 lymph nodes. No. 12 lymph node enlargement always occurs in advanced lower 1/3 GC and may lead to bile duct obstruction. Many studies have focused on No. 12 lymph node metastasis. Maruyam et al. [14] showed that the rate of No. 12 lymph node metastasis in lower 1/3 GC (6.8%) was higher than that in middle (2.6%) and upper (2.7%) 1/3 GC. A study by Feng et al. [15] also confirmed the same results, reporting 7.7% as the rate of No. 12b lymph node metastasis and 15.4% as the rate of No. 12p lymph node metastasis in lower GC. In addition, No. 12b and No. 12p lymph node metastasis is related to No. 5 and No. 12a lymph node metastasis. Interestingly, all patients with No. 12b or No. 12p lymph node metastasis had No. 5 lymph node metastasis, indicating that No. 5 lymph node metastasis may be a predictive factor for No. 12b or 12p lymph node metastasis. No additional value or improvements in the 3-year survival rate were observed in any patient with No. 12b or No. 12p lymph node metastasis. However, cT3 GC patients with No. 12b or No. 12p lymph node metastasis could obtain a survival benefit, while cT4a GC patients with No. 12b or No. 12p lymph node metastasis could not. Multivariate analysis confirmed that No. 12b and No. 12p lymph node metastasis is not an independent prognostic factor [16]. Furthermore, dissection of the No. 12p lymph nodes is beneficial for ligating the right gastric artery and dissecting the duodenum in GC with duodenal invasion.

The No. 13 lymph nodes are defined as the lymph nodes on the posterior surface of the pancreatic head cranial to the duodenal papilla. According to the new Japanese Classification of Gastric Carcinoma, the No. 13 lymph nodes are regarded as regional lymph nodes in GC with duodenal invasion. However, dissection of the No. 13 lymph nodes is also in dispute, especially in lower 1/3 GC. Because of the existence of the pyloric canal, metastasis may differ between GC invading the duodenum and GC invading the pylorus. Liang et al. [17] showed that patients with lower 1/3 GC can gain a survival benefit and additional value by undergoing D2-plus lymphadenectomy including the No. 13 lymph nodes. Our study showed a high IEBLD (10.96) due to the better 3-year OS rate (58.3%) and high metastasis rate (18.8%) of patients with No. 13 lymph node metastasis compared with the IEBLD for N2 lymph nodes (Nos. 1, 9, 11p, and 12a). We could produce a survival benefit or additional value by dissecting the No. 13 lymph nodes in not only pT3 GC patients but also pT4 GC patients. Furthermore, no obstructive jaundice caused by No. 13 lymph node metastasis after dissection of the No. 13 lymph nodes has been found in any patient during the follow-up study to date. Interestingly, there was no difference between patients with pyloric invasion (18/97) only and patients with duodenal invasion (6/31) with respect to No. 13 lymph node metastasis.

The No. 14v lymph nodes are defined as the lymph nodes along the SMV. The No. 14v lymph nodes are regarded as regional gastric lymph nodes in the new Japanese Classification of Gastric Carcinoma. However, the controversy regarding dissection of the No. 14v lymph nodes is heated. In most cases, dissection of the No. 14 lymph nodes depends on the habits of surgeons. A study by Eom et al. [18] confirmed that dissection of No. 14v is an independent prognostic factor in lower 1/3 GC, especially for stage III/IV type GC. Moreover, a study by Masuda et al. [19] showed that dissection of No. 14v could improve the 5-year OS rate of patients with No. 14v lymph node metastasis. In our present study, a considerable 3-year OS rate (53.6%) and a high rate of No. 14 lymph node metastasis (21.8%) led to the high IEBLD (11.68%) for these patients. It was common for patients (24/28) with No. 14v lymph node metastasis to also exhibit No. 6 lymph node metastasis. This finding may confirm one of the mechanisms of GC lymph node metastasis, in which the No. 14v lymph node station is downstream of the No. 6 lymph node station. However, No. 6 lymph node metastasis was not observed in 2 patients with No. 14v lymph node metastasis. Interestingly, none of these patients survived for 3 years, which may indicate that a new mechanism of lymph node metastasis is present in patients with No. 14v lymph node metastasis.

In our study, all patients successfully underwent radical gastrectomy and D2-plus lymphadenectomy, with a relatively low rate of postoperative complications (13.2%), which included incision infection (2 patients), lymphorrhagia (4 patients), anastomotic stricture (4 patients), pancreatic leakage (3 patients) and anastomotic leakage (4 patients). Despite the deep location of some of the lymph nodes included in D2-plus lymphadenectomy, no increased bleeding events were observed. Diagnosing and treating postoperative complications to avoid additional adverse sequelae is important.

In short, additional value and a 3-year survival benefit are observed with dissection of the No. 13 and No. 14v lymph nodes in GC with pyloric invasion, but there is no obvious survival benefit achieved by dissecting the No. 8p, No. 12b, or No. 12p lymph nodes in these patients. Furthermore, in some cT3 GC patients, dissection of the No. 12b and No. 12p lymph nodes can yield a survival benefit or additional value. However, this study has some limitations. First, it had only a short follow-up period, and the 5-year OS rate may be better for evaluating the therapeutic value because the IEBLD is commonly calculated by multiplying the frequency of lymph node metastasis to each station by the 5-year OS rate of patients with metastasis to that station. Second, a group of patients treated with standard D2 lymphadenectomy in GC with pyloric invasion is needed to evaluate the benefit of D2-plus lymphadenectomy in those patients.

## Conclusion

In conclusion, our results conform to the new guidelines of the JGCA. We support the proposal to regard the No. 14v lymph nodes as regional lymph nodes in the new guidelines, and no additional benefit was observed in dissecting the No. 8p, No. 12b, and No. 12p lymph nodes, which are regarded as distant lymph nodes. The No. 13 lymph nodes may be better regarded as regional lymph nodes in GC with pyloric invasion. However, we suggest that the No. 12b and No. 12p lymph nodes may be better dissected in cT3 GC patients with pyloric invasion.

## Data Availability

The datasets used and analyzed during the current study are available from the corresponding author on reasonable request.

## Abbreviations

CBD: Common bile duct
CHA: Common hepatic artery
GC: Gastric cancer
GDA: Gastroduodenal artery
GTH: Gastrointestinal vein trunk
MCV: Middle colic vein
PHA: Proper hepatic artery
PV: Portal vein
RCV: Right superior colic vein
RGEV: Right gastroepiploic vein
SMV: Superior mesenteric vein
